# Pathogenicity of an African swine fever virus strain isolated in Vietnam and alternative diagnostic specimens for early detection of viral infection

**DOI:** 10.1186/s40813-021-00215-0

**Published:** 2021-05-02

**Authors:** Hu Suk Lee, Vuong Nghia Bui, Duy Tung Dao, Ngoc Anh Bui, Thanh Duy Le, Minh Anh Kieu, Quang Huy Nguyen, Long Hoang Tran, Jae-Hee Roh, Kyoung-Min So, Tai-Young Hur, Sang-Ik Oh

**Affiliations:** 1 International Livestock Research Institute (ILRI), Hanoi, Vietnam; 2 Virology Department, National Institute of Veterinary Research, 86 Truong Chinh, Dong Da, Hanoi, Vietnam; 3 Division of Animal Disease & Health, National Institute of Animal Science, Rural Development Administration, 55365 Wanju, Republic of Korea

**Keywords:** African swine fever, Vietnam, Pathogenicity, Incubation period, Clinical signs, Virus excretion pattern, Alternative diagnostic specimen

## Abstract

**Background:**

African swine fever (ASF), caused by the ASF virus (ASFV), was first reported in Vietnam in 2019 and spread rapidly thereafter. Better insights into ASFV characteristics and early detection by surveillance could help control its spread. However, the pathogenicity and methods for early detection of ASFV isolates from Vietnam have not been established. Therefore, we investigated the pathogenicity of ASFV and explored alternative sampling methods for early detection.

**Results:**

Ten pigs were intramuscularly inoculated with an ASFV strain from Vietnam (titer, 10^3.5^ HAD_50_/mL), and their temperature, clinical signs, and virus excretion patterns were recorded. In addition, herd and environmental samples were collected daily. The pigs died 5–8 days-post-inoculation (dpi), and the incubation period was 3.7 ± 0.5 dpi. ASFV genome was first detected in the blood (2.2 ± 0.8) and then in rectal (3.1 ± 0.7), nasal (3.2 ± 0.4), and oral (3.6 ± 0.7 dpi) swab samples. ASFV was detected in oral fluid samples collected using a chewed rope from 3 dpi. The liver showed the highest viral loads, and ear tissue also exhibited high viral loads among 11 tissues obtained from dead pigs. Overall, ASFV from Vietnam was classified as peracute to acute form. The rope-based oral fluid collection method could be useful for early ASFV detection and allows successful ASF surveillance in large pig farms. Furthermore, ear tissue samples might be a simple alternative specimen for diagnosing ASF infection in dead pigs.

**Conclusions:**

Our data provide valuable insights into the characteristics of a typical ASFV strain isolated in Vietnam and suggest an alternative, non-invasive specimen collection strategy for early detection.

## Background

African swine fever (ASF) is a highly contagious hemorrhagic viral disease that affects domestic and wild pigs in all age groups, causing substantial economic and production losses [1]. ASF has been included in the list of notifiable diseases by the World Organization for Animal Health (OIE) [1]. ASF is caused by ASF virus (ASFV), a double-stranded DNA virus of the family *Asfaviridae* and the genus *Asfivirus* and having a diameter of 175–215 nm [2]. The virus contains 151–167 genes in its 170–192 kbp long genome [2, 3], and a total of 24 genotypes have been identified worldwide [4, 5].

The virulence of different ASFV isolates in the same host may vary, and the ASF outbreaks in Asia have been caused by highly virulent strains [6]. These highly virulent ASFV strains caused mortality in 90–100 % of cases. A previous study reported that the Chinese ASFV isolates are highly lethal to domestic pigs even at low doses of the virus and that the clinical signs start showing very early period during the course of infection [6]. A recent study revealed that a 10-nucleotide insertion (5′-GGAATATATA-3′) was detected in all Vietnamese ASFV isolates, similar to that detected in the ASFV isolates from China [7]. In Vietnam, the first ASF outbreak was detected in February 2019 at a family-owned backyard farm in Hun Yen province. The pig farm was situated approximately 50 km south of Hanoi and about 250 km from the China border [8]. Since then, the virus has rapidly spread across the country, leading to the culling of approximately six million pigs (20 % of Vietnam’s pig production) [9]. As of November 2020, ASF outbreaks have been reported in more than 20 of the country’s 63 provinces [9]. Several molecular and pathological studies on ASFV-infected pigs from ASF outbreak farms were conducted to evaluate the characteristic lesions and examine the genetic properties of ASFV strains from Vietnam [10–12]. Because no virus inoculation studies have been conducted to evaluate the virulence and pathogenicity of ASFV isolates from Vietnam, the pathogenicity of these strains is not fully understood. To address these knowledge gaps, we investigated the virulence and pathogenicity of ASFV strains from Vietnam by experimentally challenging pigs with ASFV.

Given that there is no effective vaccine against ASFV, the best control method for this virus is implementing strict prevention measures and early detection of the ASF-suspected pigs on farms. However, ASF is very difficult to differentiate from other pig diseases because clinical signs and lesions observed in post-mortem examinations are very similar [13–15]. In particular, the well-known pig diseases such as classical swine fever (CSF) and highly pathogenic porcine reproductive and respiratory syndrome (PRRS), which are endemic in Vietnam, show very similar hemorrhagic lesions in the lymph nodes [16, 17]. The gold standard of ASFV diagnosis is virus isolation from organs, and its surveillance is commonly performed using blood samples [1]. However, blood sampling and testing procedures are time-consuming and not suitable for on-site pig farm use. This is because the virus could be spread between farms or within farms by the person who collects blood samples for ASF surveillance. Moreover, it is particularly not useful for farms in less developed regions where limited veterinary services are available. For this reason, finding more suitable specimen types that would provide an efficient and cost-effective alternative to blood/serum for the detection of ASFV genetic material is essential.

Therefore, this study’s main objective was to evaluate incubation time, clinical signs, viremia, and viral loads in different samples using the time-series analysis of ASFV-inoculated pigs. We also sought to identify a simple, non-invasive specimen type for early detection of ASF-suspected pigs on farms. This study’s outcomes offer novel insights into the virulence and pathogenicity of a typical ASFV strain from Vietnam and propose a viable, non-invasive alternative to serum to allow early detection of ASFV.

## Results

### Clinical assessment

**Fig. 1:**
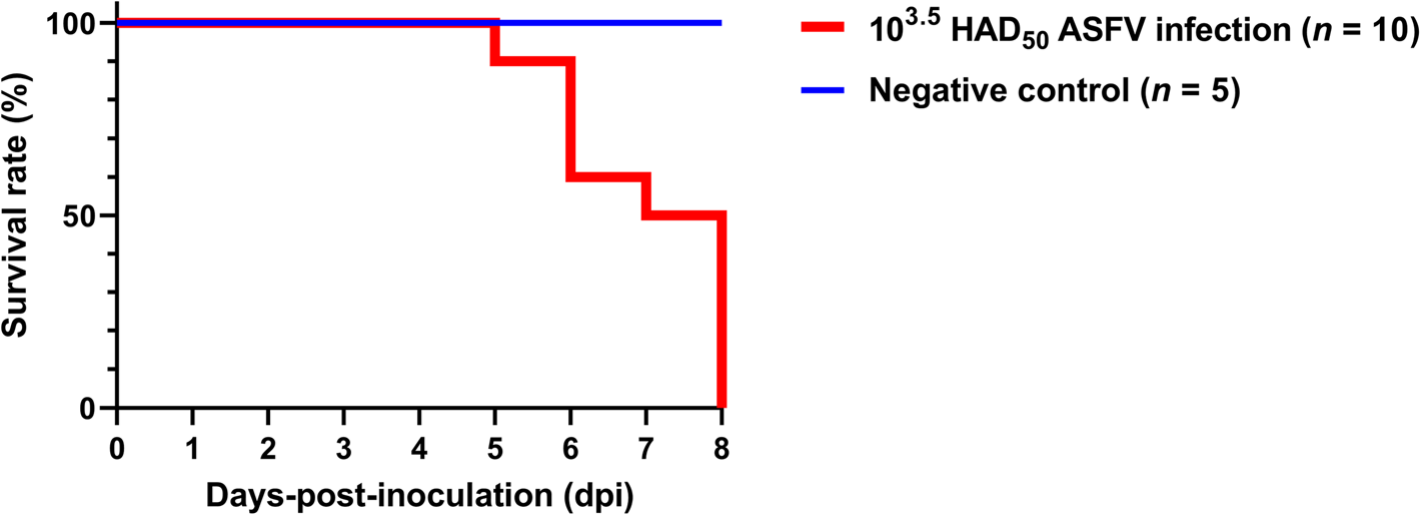
Pig survival rates in the ASFV infection (red line) and negative control (blue line) groups

**Fig. 2:**
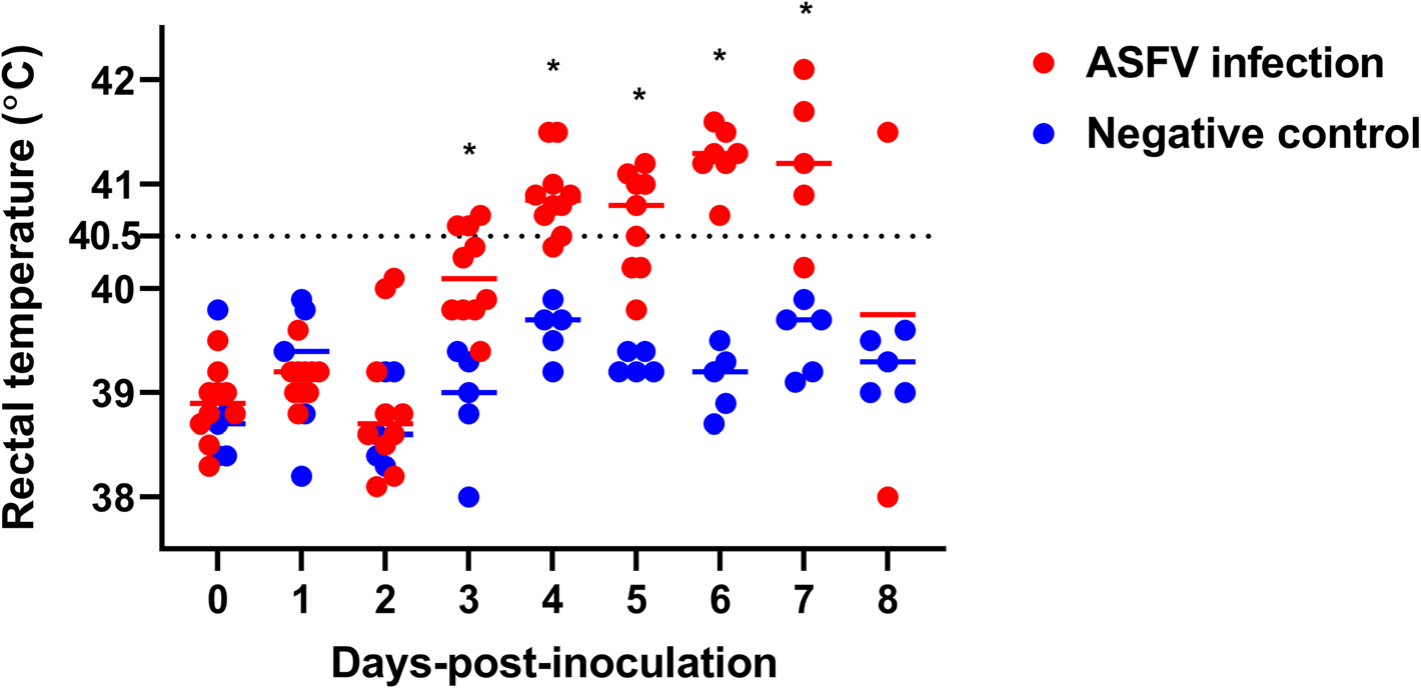
Individual rectal temperatures in pigs from the ASFV infection and negative control groups. Red and blue circles and horizontal lines indicate individual and mean temperatures of the ASFV and negative control groups, respectively. Red and blue short lines represent the average temperature of ASFV infection and negative control group, respectively, at each time point. * *P* < 0.05

Experimentally infected pigs died within 5–8 days-post-inoculation (dpi), as shown in Fig. 1. On average, pig death occurred at 7.0 ± 1.2 dpi. In the ASFV infection group, three pigs started to show high fever (> 40.5 ℃) at 3 dpi, and their mean temperature readings were 40.9 ± 0.3 (4 dpi), 40.6 ± 0.5 (5 dpi), 41.3 ± 0.3 (6 dpi), 41.2 ± 0.6 (7 dpi), and 39.8 ± 1.8 ℃ (8 dpi) (Fig. 2). As expected, the pigs from the negative control (NC) group showed normal temperature during the study period. We observed a significant effect of time (dpi) on the mean rectal temperatures (*P* < 0.001) and a significant group effect between the two experimental groups (*P* = 0.009). Furthermore, a significant time-by-group interaction was noted for the rectal temperature in the two groups (*P* < 0.001). The mean temperatures for the two groups were not statistically different until 2 dpi, whereas the temperatures differed significantly between the two groups (*P* < 0.05) from 3 to 7 dpi.

**Fig. 3:**
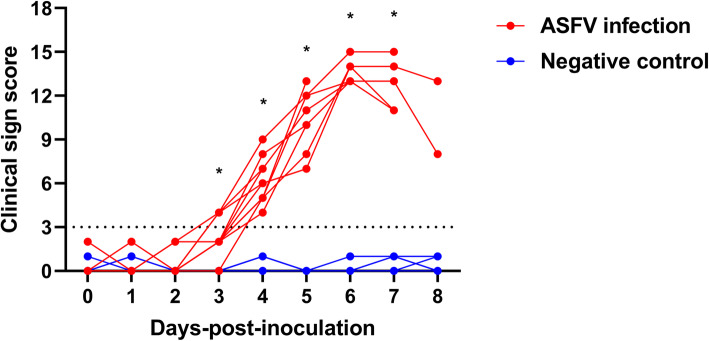
Clinical scores of pigs from the ASFV (red lines) and negative control (blue lines) groups. The scores were calculated based on the protocol published by Galindo-Cardiel et al. [18] with minor modifications (Table 2). * *P* < 0.05

The time-course changes in clinical sign scores of the two groups are shown in Fig. 3. Only one pig presented behavioral changes at 1 dpi, namely, decreased activity and mild to moderate clumsiness. However, digestive/respiratory symptoms were observed in all pigs in the ASFV infection group at 4 dpi. At 5 dpi, four pigs (44.4 %) in the ASFV infection group reached a clinical sign score > 10, and all were dead at 8 dpi. Cutaneous ecchymosis on the legs and abdomen was observed in two ASFV-infected pigs on the days preceding death (5 and 8 dpi, respectively). By contrast, no clinical signs were observed in the NC group. Changes in clinical sign scores were significantly different between the two groups (*P* < 0.001), and inter-group comparisons also revealed a significant difference over time (*P* < 0.001). The mean clinical sign scores for the two groups were significantly (*P* < 0.05) different between 3 and 7 dpi. The mean clinical sign scores were as follows: 0.2 ± 0.6 (1 dpi), 0.4 ± 0.8 (2 dpi), 2.4 ± 1.3 (3 dpi), 6.2 ± 1.5 (4 dpi), 10.0 ± 2.2 (5 dpi), 13.7 ± 0.8 (6 dpi), 12.8 ± 1.8 (7 dpi), and 12.0 ± 5.7 scores (8 dpi). These results indicated that the inoculated pigs first started to show clinical signs at 3.7 ± 0.5 dpi.

### Detection of ASFV genome in various specimens

We calculated the mean ASFV load in various samples in the three types of specimens, namely individual pig samples, herd samples, and environmental samples.

**Fig. 4:**
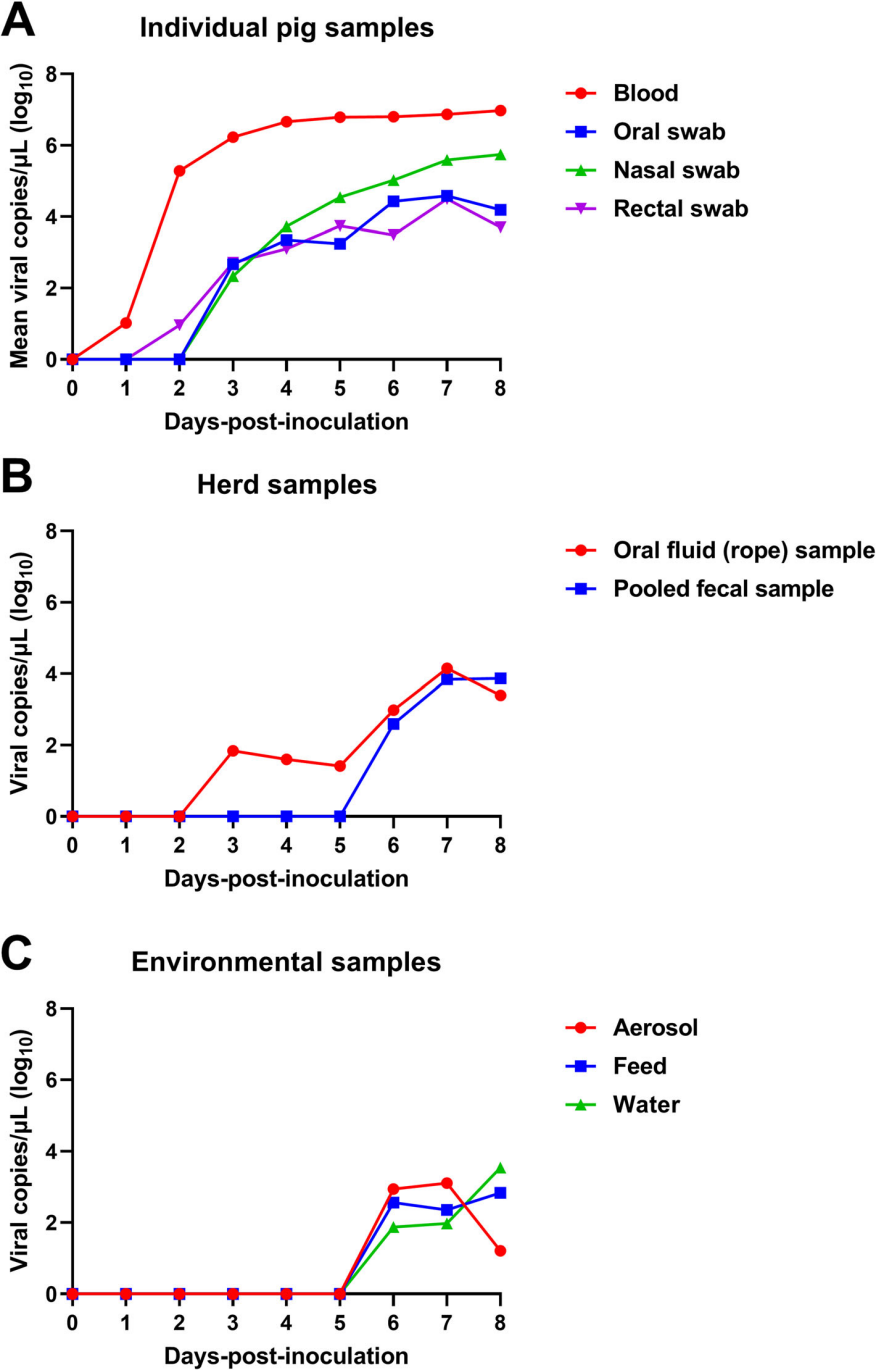
Patterns of ASFV excretion observed in experimentally infected pigs. Mean viral copy numbers per 1 µL in (**a**) individual ASFV-infected pig samples (blood, oral swab, nasal swab, and rectal swab), (**b**) ASFV-infected herd samples (oral fluids collected using the rope-based method and pooled fecal samples), and (**c**) environmental samples of the ASFV-infected herd (aerosol, feed, and water)

In the individual pig samples, ASFV genomic DNA was first detected in the blood at 2.2 ± 0.8 dpi (range 1–4 dpi), and then in the rectal (3.1 ± 0.7 dpi; range 2–4 dpi), nasal (3.2 ± 0.4 dpi; range 3–4 dpi), and oral (3.6 ± 0.7 dpi; range 3–5 dpi) swab samples. The average viral loads from samples per dpi are shown in Fig. 4a. In particular, we observed a relatively low viral load (1.1 × 10^2^ copies/µL) in blood samples at 1 dpi in one ASFV-infected pig, and 5.3 × 10^1^ and 3.9 × 10^1^ copies of viral genomes per 1 µL were detected in the rectal swab samples of two pigs at 2 dpi. ASFV p72 gene was observed for the first time at 3 dpi in eight nasal swabs (average: 1.7 × 10^2^ copies/µL) and five oral swab samples (average: 2.3 × 10^2^ copies/µL), respectively. At 8 dpi, in the last pigs that died on the day, the average titer was the highest in blood samples (9.5 × 10^6^ copies/µL), followed by the nasal (5.5 × 10^5^ copies/µL), oral (1.6 × 10^4^ copies/µL), and rectal (5.1 × 10^3^ copies/µL) swab samples.

The viral loads in herd samples (oral fluids collected by the rope-based method and pooled fecal samples) from the ASFV infection group are shown in Fig. 4b. ASFV genomic DNA was first observed in the oral fluid samples at 3 dpi (6.9 × 10^1^ copies/µL); the loads recorded after that were as follows: 4.0. × 10^1^ (4 dpi), 2.6 × 10^1^ (5 dpi), 9.6 × 10^2^ (6 dpi), 1.4 × 10^4^ (7 dpi), and 2.5 × 10^3^ copies/µL (8 dpi). In the pooled fecal samples, the viral genomic DNA was detected later, at 6, 7, and 8 dpi with loads of 3.9 × 10^2^, 7.0 × 10^3^, and 7.4 × 10^3^ copies/µL, respectively.

The time-course of viral loads in the three environmental samples (aerosol, feed, and water) is shown in Fig. 4c. All three types of environmental samples harbored ASFV p72 DNA from 6 dpi (aerosol, 8.8 × 10^2^ copies/µL; feed, 3.7 × 10^2^ copies/µL; water, 7.5 × 10^1^ copies/µL). The viral loads in the feed and water samples gradually increased over time, whereas viral loads in the aerosol samples dramatically decreased between 7 (1.3 × 10^3^ copies/µL) and 8 dpi (1.6 × 10^1^ copies/µL).

ASFV was not detected in any sample from the NC group during the study period.

### Gross pathological findings

Hemorrhagic edema of lymph nodes (mesenteric, inguinal, and submaxillary), splenomegaly, and enlarged liver were observed in all 10 ASFV-infected pigs. Nine pigs showed hemorrhagic pulmonary edema, and eight pigs had enlarged kidneys with swelling edema. In addition, one pig showed petechial hemorrhages in the renal cortex. We observed the accumulation of yellowish fluids in the pericardial cavity (hydropericardium) in seven pigs and hemorrhagic lesions in the colon of three ASFV-infected pigs.

### Virus replication in tissues of dead pigs

All infected pigs were identified as being positive for the presence of AFSV using qPCR in the tested organs, namely, the liver, spleen, inguinal/mesenteric/submandibular lymph nodes, tonsils, heart, lungs, kidneys, colon, and ear tissues (Table 1). The highest mean viral genome loads were observed in the liver (1.5 × 10^6^ copies/µL), followed by those in the spleen (6.5 × 10^5^ copies/µL) and inguinal lymph node (2.9 × 10^5^ copies/µL). By contrast, the lowest mean viral loads were detected in the kidney (6.3 × 10^4^ copies/µL), followed by those in the heart (9.9 × 10^4^ copies/µL) and colon (1.6 × 10^5^ copies/µL). Notably, qPCR revealed that ear tissue samples from 10 dead pigs contained—on average—2.5 × 10^5^ copies/µL of the ASFV p72 gene, a value that was higher than that observed in the tonsil (2.2 × 10^4^ copies/µL), colon (1.6 × 10^5^ copies/µL), heart (9.9 × 10^4^ copies/µL), and kidney samples (6.3 × 10^4^ copies/µL). The highest viral load (7.8 × 10^6^ copies/µL) was recorded in the liver tissue of the pig that died at 8 dpi, followed by the spleen of the pig that died at 5 dpi (1.4 × 10^6^ copies/µL). The lowest viral load (1.1 × 10^3^ copies/µL) was found in the spleen tissue of another pig, which also showed the lowest viral loads in the liver (4.2 × 10^4^ copies/µL), kidney (1.9 × 10^4^ copies/µL), and colon tissue (2.0 × 10^4^ copies/µL). However, the ear tissue sample from this pig presented the highest viral load (1.2 × 10^6^ copies/µL) among all ear tissue samples tested.

**Table 1:** Replication of the African swine fever virus strain (VNUA/HY/Vietnam) in infected pigs

Viral load	Virus titer (copy numbers of the viral p72 gene) in tissues of dead pigs (*n* = 10)										
	Liver	Spleen	Lymph node			Tonsil	Heart	Lung	Kidney	Colon	Ear tissue
			Inguinal	Mesenteric	Submandibular						
Mean ± SD	1.5 × 10^6^ ± 2.4 × 10^6^	6.5 × 10^5^ ± 4.1 × 10^5^	2.9 × 10^5^ ± 2.8 × 10^5^	2.9 × 10^5^ ± 1.9 × 10^5^	2.8 × 10^5^ ± 1.5 × 10^5^	2.2 × 10^5^ ± 2.1 × 10^5^	9.9 × 10^4^ ± 1.6 × 10^5^	2.9 × 10^5^ ± 2.0 × 10^5^	6.3 × 10^4^ ± 7.0 × 10^4^	1.6 × 10^5^ ± 3.0 × 10^5^	2.5 × 10^5^ ± 3.3 × 10^5^
Highest	7.8 × 10^6^	1.4 × 10^6^	1.1 × 10^6^	6.5 × 10^5^	5.2 × 10^5^	6.8 × 10^5^	5.6 × 10^5^	6.4 × 10^5^	2.6 × 10^5^	1.0 × 10^6^	1.2 × 10^6^
Lowest	4.2 × 10^4^	1.1 × 10^3^	4.8 × 10^4^	8.3 × 10^4^	1.1 × 10^4^	1.9 × 10^4^	1.1 × 10^4^	8.2 × 10^4^	1.9 × 10^4^	2.0 × 10^4^	1.7 × 10^4^

## Discussion

Given the variable pathogenicity of ASFV—ranging from acute to chronic disease depending on the strain—the characterization of field virus isolates is essential for controlling and managing the ASF epidemic. The current study aimed to obtain information about the VNUA/HY/Vietnam ASFV strain pathogenicity and its excretion pattern in various samples. To the best of our knowledge, this is the first experimental viral infection study to evaluate the incubation time, clinical signs, viremia, and virus excretion patterns in pigs infected with an ASFV isolated in Vietnam that belongs to p72 genotype II [8]. In this animal experiment, we also investigated the potential risk factors associated with ASFV infection of farm pigs and their environmental samples, allowing ASFV transmission within and between pens. Moreover, we explored alternative specimens to enable farmers to identify ASFV-infected pigs early and facilitate viral surveillance.

The incubation period of ASFV, ranging between 3 and 19 days depending on the strain, inoculation dose, route of transmission, and host characteristics [13, 19, 20], is one of the most critical epidemiological parameters for evaluating ASFV pathogenicity [21]. In the present study, the incubation period of the VNUA/HA/Vietnam ASFV strain injected intramuscularly 1 mL of the virus at 10^3.5^ 50 % hemadsorbing doses (10^3.5^ HAD_50_/mL) was 3.7 ± 0.5 days, which was similar to the incubation period of the Pig/HLJ/18 strain from China (3–5 dpi) [6]. The incubation period in this study was shorter than that noted for the Georgia 2007/1 strain (5.6 ± 0.8 days) and Chechen Republic 2009 strain (4.4 ± 1.0 days) administered at a dose of 10^3^ HAD_50_/mL [22, 23]. All infected pigs in the current study died within 5–8 dpi (7.0 ± 1.2 days), earlier than the lethality observed after virus inoculations with the strains from China (8.0 ± 1.4), Georgia (8.8 ± 1.1), or the Chechen Republic (9.2 ± 0.8 days) [22–24]. Although the incubation and death periods, as well as clinical presentation of the ASFV strain in this study, were compatible with those of both peracute or acute form disease, the clinical courses of the infected pigs and post-mortem data, including pulmonary edema, multiorgan hemorrhages, hyperemic splenomegaly, and hemorrhagic lymph nodes, strongly suggested acute form of the disease. These post-mortem findings were consistent with other field studies in farms affected by the ASF outbreak in Vietnam [12, 18]. Overall, the VNUA/HA/Vietnam ASFV strain investigated in this study caused peracute to an acute form of ASF based on the short incubation and early death period, clinical presentation, and various gross pathological findings [1, 25].

This study indicated that the within-pen transmission of ASFV could occur at a very early stage of infection through various routes. The viral genomic DNA was detected in blood samples at 2.2 ± 0.8 dpi. Notably, 80 % of the pigs in the ASFV infection group excreted the virus already by 2 dpi, indicating that the virus could spread rapidly within pens before the clinical signs become evident. After 3.7 ± 0.5 dpi, the numbers of ASFV genome copies dramatically increased from 4.5 × 10^6^ copies/µL (4 dpi) to 9.5 × 10^6^ copies/µL (8 dpi) in blood samples. This result was in agreement with data from previous studies [6, 13, 21] and supported the notion that blood acts as the most important vehicle for virus transmission to other pens or farms. In Vietnam, clotted blood and organs are commonly collected from slaughtered pigs for human consumption or used as pig feed. When blood is collected from ASFV-infected animals and is not properly inactivated, it could facilitate ASFV transmission between pens and farms. Further, ASFV genomic DNA was detected at lower levels in oral, nasal, and rectal swab samples than in the blood. Although viral DNA was detected earlier in the rectal swabs (2 dpi) than in both oral and nasal swabs (3 dpi), the numbers of viral copies in rectal swab samples (4.6 × 10^1^ copies/µL) were too low for the latter to serve as a reliable diagnostic specimen. The copy numbers of viral genes were higher in the oral and nasal swab samples than in the rectal swab samples collected later in the incubation period (4–8 dpi). These results suggested that the oronasal samples would be relevant specimens for the early detection of ASFV-infected pigs, consistent with reports for the PRRS and CSF viruses [26, 27].

The spread of ASF can only be controlled by early detection, meticulous surveillance, strict biosecurity measures, and animal movement control [28, 29]. To perform successful ASF surveillance in farms, it is necessary to obtain relevant blood specimens from pigs with suspected ASFV infection. However, the identification of such pigs is difficult because of the short incubation period. The appropriate surveillance might not succeed, especially in developing countries, due to the limited availability of veterinary services [30]. The time lag to initiate sampling enabled ASF outbreaks to rapidly spread across these countries.

For this reason, we tried to identify a non-invasive surveillance specimen that could serve as an alternative to blood samples for early ASFV detection. Interestingly, the current findings suggested that ASFV could be detected relatively early in oral fluid samples collected using the rope-based method (ASFV was detected in our samples from an ASFV-infected herd at 3 dpi), consistent with the results of a previous study [31]. The rope-based sampling technique could be useful for the early detection of ASFV in domestic pigs at the herd level. It is effortless and convenient for livestock farmers in rural areas, especially in remote mountainous/areas where animal health professionals are not immediately available for sampling. Moreover, this alternative non-invasive specimen would allow successful ASF surveillance on large pig farms. In addition, it should be noted that ASFV was detectable in pooled feces on the floor from 6 dpi, implying that the virus shedding begins at approximately the same time as the onset of death. This finding contrasted the results of a previous study where ASFV shedding began with fever onset [32]. Moreover, the time at which ASFV was first detected was inconsistent between rectal swabs (2–4 dpi) and pooled feces (6 dpi) samples. This discrepancy could be due to the very low ASFV load in the pooled feces at the early time points (e.g., the number of viral copies in the rectal swab was only 4.6 × 10^1^ copies/µL at 2 dpi). Nevertheless, this result provided valuable information that the infected feces could act as an ASFV vehicle to easily contaminate the feed and water sources for other pigs in the same pen.

In air samples, viral DNA was also observed at 6 dpi, the same time point when ASFV genes appeared in the pooled fecal samples. Transmission of ASFV via air is not thought to play a significant role in the introduction of the virus into pig herds [33], but several previous studies suggested that ASFV could be transmitted by aerosol over short distances and for very short periods (less than 1 h) [34–36]. Our results provide evidence supporting the previously proposed hypothesis that the transmission of ASFV via air can occur within a closed environment and that the amounts of virus in the aerosol are positively associated with their levels in feces. The feed and water could be contaminated by ASFV within 6 days after the herd became infected, demonstrating that the virus might be transmitted within and between farms via humans or feed delivery at an early stage of infection. It is currently difficult to establish the viral titer needed for an infection; further studies would be needed to evaluate the minimum dose of ASFV that could be transmitted in various scenarios.

According to the OIE report, ASFV could be diagnosed in necropsied pigs by sampling spleen and lymph nodes, which usually contain the highest viral loads [1]. However, our measurements of the viral loads in dead pigs suggested that the liver is one of the most valuable organs in the post-mortem diagnosis of ASF. The liver showed the highest viral loads (average: 1.5 × 10^6^ copies/µL) among the 11 sampled organs and tissue, and it can be easily sampled during necropsy compared to organs such as the lymph nodes or tonsils. Nevertheless, the necropsy procedure and organ sampling in ASFV-infected pigs directly on the farm field creates a possibility of virus leakage within the same herd or into the environment. Therefore, we investigated viral loads in ear tissue samples, which could be easily obtained using an ear-notcher by the farmers themselves. Although the ear tissue samples showed relatively lower viral copy numbers (2.5 × 10^5^ copies/µL) than the liver (1.5 × 10^6^ copies/µL) and spleen (6.5 × 10^5^ copies/µL), its viral load was higher than that of the tonsil samples (2.2 × 10^5^ copies/µL). Furthermore, the lowest viral load in the ear tissue (1.7 × 10^4^ copies/µL) in the cohort of 10 ASFV-infected pigs was still higher than that in the spleen (1.1 × 10^3^ copies/µL), implying that the former specimen could be useful for ASF diagnosis in dead pigs without a necropsy. This approach has the advantage of preventing virus leakage in farms. It is particularly useful for farms in rural areas where veterinary services are not readily available because only samples from the ear tissue of dead pigs would be necessary to confirm ASFV infection in the laboratory.

## Conclusions

This study provides valuable information about an endogenous ASFV strain from Vietnam, specifically about its incubation period, clinical signs, pathogenicity, and viral loads in different organs and tissues, as well as in environmental samples. The virulence of the ASFV strain examined in this study was classified as “from peracute to acute form” The oral and nasal routes are important for ASFV transmission within identical herds, and oral fluid samples collected using the rope-based method were useful for detecting ASFV. In addition, our study found that ASFV could be detected in several environmental samples (e.g., aerosol, feed, and water) in the limited space where the animal experiments were conducted, implying that these samples might be potential risk factors for ASFV transmission between pigs. The ear tissue—cut using an ear-notcher from dead pigs—may be a simple alternative specimen for the detection of ASFV. Overall, this study provides invaluable information regarding the characteristics of ASFV in Vietnam and provides an alternative, non-invasive sampling method to facilitate early detection and effective surveillance.

## Methods

### Animals

A total of 15 pigs (Yorkshire × Landrace × Duroc) aged 7–8 weeks were obtained from the same herd from a commercial pig farm. All pigs tested negative for the five specific pathogens: foot-and-mouth disease virus, porcine circovirus 2, PRRS virus, CSF virus, and ASFV. The pigs were also carefully inspected by veterinary researchers before entering the laboratory facility. All healthy pigs were introduced into the biosafety facility of the National Institute of Veterinary Research (NIVR) in Vietnam one week before being inoculated with the virus. The pigs were fed a commercial diet twice daily, and water was provided *at libitum*. Room temperature and humidity at the biosafety facility were recorded daily.

### Virus strain

The virus strain used in this study was the Vietnamese ASFV isolate VNUA/HY/Vietnam (GenBank accession no. MK554698), which is genetically very close to ASFV isolates that are currently in circulation in Asia. This virus strain was provided by Associate Professor Le Van Phan, Vietnam National University of Agriculture. The virus was propagated in primary porcine alveolar macrophages in Dulbecco’s modified Eagle medium supplemented with 5 % fetal bovine serum and stored at − 80 ℃ until use. The VNUA/HY/Vietnam virus was quantified using the hemadsorption (HAD) assay as described previously [37] with minor modification. Primary porcine alveolar cells were seeded in a 96-well cell culture plate and titrated using a 10-fold serial dilution. After seven days of culture, the HAD_50_/mL was calculated as described previously [38].

### Experimental infection in animals

 The animal experiments performed within the biosafety facility at the NIVR in Vietnam followed the guidelines approved by the Institutional Animal Care and Use Committee at the National Institute of Animal Science in South Korea and complied with NIVR guidelines. All pigs were randomly divided into two groups [ASFV infection group, *n* = 10; NC group, *n* = 5] and housed in two isolated biosafety facilities (Animal biosecurity level 2 enhanced facility). A total of 10 pigs were intramuscularly inoculated with ASFV at a titer of 10^3.5^ HAD_50_/mL. The remaining five pigs were treated as the mock infection group. During the experiments, the room temperature was 27–29 °C, and the relative humidity ranged from 65 to 70 % for both the ASFV infection and NC groups. Clinical signs and rectal temperature were recorded at regular daily intervals from the day of virus inoculation. The clinical signs were scored as described in the previous report with minor modifications [18] (Table 2).

**Table 2:** Clinical signs used to calculate African swine fever clinical sign scores. The list was generated based on the protocol published by Galindo-Cardiel et al. [18] with minor modifications.

Clinical signs	Scores (0: normal, 1: mild, 2: moderate, and 3: severe)
Fever	0: < 39.5 °C 1: 39.5–40.5 °C 2: 40.6–41.0 °C 3: > 41.0 °C
Behavior	0: Normal 1: Decreased activity, mild to moderate clumsiness 2: Decreased external stimuli response 3: Markedly decreased or lack of external stimuli response, immobile, prostration
Skin	0: Normal 1: Body cyanotic areas (< 10%); minimal multifocal cutaneous necrosis and/or hemorrhages 2: Body cyanotic areas (11-25%); mild to moderate multifocal cutaneous necrosis and/or hemorrhages 3: Body cyanotic areas (> 25%); moderate to marked multifocal cutaneous necrosis and/or hemorrhages
Digestive system	0: Normal 1: Feces around the anus (mild diarrhea) 2: Feces covering posterior gluteus (moderate diarrhea) 3: Feces covering posterior gluteus with blood or extensive mucus (severe bloody diarrhea)
Respiratory system	0: Normal 1: Mild dyspnea (labored respiration) 2: Moderate dyspnea or cough 3: Marked dyspnea or severe labored respiration
Body condition	0: Normal, full stomach 1: Empty stomach, sunken flanks 2: Empty stomach, sunken flanks, loss of muscle mass 3: Emaciated
Total	From 0 to 18 (over than three scores considered as the pigs showing clinical signs)

### Collection of samples

To analyze the virus excretion patterns and identify potential alternative methods for obtaining diagnostic specimens, we collected three types of specimens from the ASFV infection group: individual pig samples, herd samples, and environmental samples. First, blood and swab (oral, nasal, and rectal) samples were collected from each pig to assess the viral DNA load by quantitative PCR (qPCR). Second, oral fluid samples were collected using the cotton rope chewing method, and pooled fecal samples were also obtained. A rope-based oral fluid sampling was performed according to the procedure described by Mur et al. [39] with a slight modification. Briefly, pigs were allowed to chew the rope for 45 min, i.e., until the rope was sufficiently wet. The wet rope was inserted into plastic bags and compressed to recover the oral fluid. Then, ~ 2.5 mL of the oral fluid was finally transferred into a 15 mL tube. Third, environmental samples (e.g., aerosol, feed, and water) from the ASFV infection group were collected for ASFV detection. The aerosol samples were obtained as described in our previous study [40].

Necropsy was conducted immediately after death by designated veterinarians, and samples of 11 organs and tissues (liver, spleen, inguinal/mesenteric/submandibular lymph nodes, lung, tonsil, colon, heart, kidney, and ear tissue) were collected for determining the number of ASFV genome copies. The ear tissue sample was collected by applying a stainless steel “V” pig ear-notcher (6 mm wide × 14 mm deep) at the dorsal edge of the ear.

### Viral gene (p72) detection by qPCR analysis

Tubes containing swab samples in the M199 medium were vortexed well to ensure mixing, and then swabs were removed from the medium. All samples were homogenized and diluted in the virus transport medium at 1:10. According to the manufacturer’s instruction (Qiagen, Hilden, Germany), viral DNA was extracted from 200 µL of the medium, whole blood, and tissue homogenates. Extracted DNA was tested for the presence of ASFV DNA by qPCR using a VDx ASFV qPCR kit (Median Diagnostics, Chuncheon, Korea). DNA (5 µL) was added into a tube containing 10 µL of the 2× master mix and 5 µL of the 4× oligo mix. The tube was placed into an IQ5 Multicolor Real-Time PCR Detection System (Bio-Rad Laboratories Ltd., Hercules, CA, USA), and the reaction proceeded under the following conditions: step 1, 50 °C for 2 min and 95 °C for 10 min; step 2, 40 cycles of 95 °C for 15 s and 58 °C for 60 s. Any samples with a cycle threshold value less than 40 were considered positive for ASFV, and the number of DNA copies was calculated based on standard samples provided by the manufacturer.

### Statistical analysis

Statistical analyses were performed using SPSS version 25.0 (IBM, Armonk, NY, USA). A linear mixed-effect model for repeated measures was used to assess determinants of the time-course of the rectal temperatures and clinical sign scores with respect to experimental groups. In this model, the experimental groups (ASFV infection and NC group), rectal temperatures, and clinical sign scores were included as fixed factors, and time (dpi) was a random variable. The mean differences in the rectal temperature and clinical sign scores were analyzed with respect to the two groups and time and an additional group-by-time interaction term. If the group × time interaction in the mixed model was significant, we then conducted Student’s *t-*tests to compare the mean values of temperature and clinical sign score between two experimental groups at the same time point. Results with *P* < 0.05 were considered significant. Data for the two groups are presented as the mean ± standard deviation calculated based on daily measurements in individual pigs.

## Data Availability

The dataset generated in this study is available from the first author and corresponding author on reasonable request.

## Abbreviations

ASF: African swine fever
ASFV: African swine fever virus
OIE: World Organization for Animal Health
CSF: Classic swine fever
PRRS: Porcine reproductive and respiratory syndrome
NIVR: National Institute of Veterinary Research
NC: Negative control
HAD_50_/mL: 50 % hemadsorbing dose per mL
dpi: Days post-inoculation
qPCR: Quantitative PCR
